# Effect of periodontitis on the development of osteoporosis: results from a nationwide population-based cohort study (2003–2013)

**DOI:** 10.1186/s12905-017-0440-9

**Published:** 2017-09-11

**Authors:** Jung-Kyu Choi, Young-Taek Kim, Hye-In Kweon, Eun-Cheol Park, Seong-Ho Choi, Jae-Hong Lee

**Affiliations:** 1 Department of Periodontology, Yonsei University College of Dentistry, Seoul, Korea; 2 Department of Periodontology, National Health Insurance Service Ilsan Hospital, Goyang, Korea; 3 Department of Preventive Medicine and Institute of Health Services Research, Yonsei University College of Medicine, Seoul, Korea; 4 Institute of Health Services Research, Yonsei University College of Medicine, Seoul, Korea; 5 Department of Periodontology, Yonsei University College of Dentistry, 50 Yonsei-ro, Seodaemun-gu, Seoul, 03722 Korea; 6 Department of Periodontology, Wonkwang University Daejeon Dental Hospital, Wonkwang University College of Dentistry, 77, Dunsan-ro, Seo-gu, Daejeon, 35233 Korea

**Keywords:** Osteoporosis, Periodontitis, Cohort study, Female, Middle-aged

## Abstract

**Background:**

The prevalence of osteoporosis associated with the aging process is anticipated to increase along with the rising aging population. Periodontitis that the most common chronic infections of humankind is considered the risk factor for osteoporosis. The aim of this study was to identify the association between osteoporosis and periodontitis using a population-based cohort.

**Methods:**

The case group was defined as patients diagnosed with periodontitis and treated with subgingival curettage, root conditioning, periodontal flap operation, bone grafting for alveolar bone defects, and guided tissue regeneration. Case and control groups matched for gender, age, household income, type of social security, disability, and residential area were generated. A Cox proportional hazard model was constructed to examine the difference in the development of osteoporosis between the case and control groups. The final sample included 13,464 participants.

**Results:**

The incidence of osteoporosis was 1.1% in males and 15.8% in females during a 10-year period. The risk factors for osteoporosis in males were increasing age and Charlson Comorbidity Index score. Periodontitis was not associated with the development of osteoporosis in males. The risk factors for osteoporosis in females were increasing age, body mass index, Charlson Comorbidity Index score, diabetes, and periodontitis. Women with periodontitis were more likely to also develop osteoporosis (HR: 1.22, 95% CI: 1.01–1.48).

**Conclusions:**

Periodontitis has an effect on the development of osteoporosis in females. Managing good teeth is required for the prevention and delay of osteoporosis. This includes dental examinations, regular cleanings and gum treatment.

## Background

Osteoporosis is a common disease related to fractures that occur at multiple skeletal sites. It causes significant morbidity and mortality [1] and is the most common reason for a broken bone among the elderly. Osteoporosis has no symptoms; it is defined as a deterioration of bone tissues and is a skeletal disorder characterized by low bone mass, which is a risk factor for an increased incidence of fracture [2, 3]. In females, bone loss increases after menopause due to lower levels of estrogen. The most common locations for osteoporotic fractures are the hip, wrist and spine.

The prevalence of osteoporosis associated with the aging process is anticipated to increase along with the increasing age of the general population. The crude prevalence rates of osteoporosis in both males and females aged 40–79 were 13.1% and 24.3%, respectively, in Korea [4]. In addition, osteoporosis is associated with a reduced health-related quality of life [5].

Periodontal diseases are closely associated with other non-communicable diseases and are the most common diseases across the globe [6]. Periodontal disease itself is caused by infection and inflammation inside the mouth after plaque has been left on the teeth for far too long. The condition occurs in 5–20% of the adult population in both developed and developing countries [7–11]. In Korea, the overall prevalence rate of periodontitis was 31.3% [12, 13]. In 2014, 24.9% of the Korean population received treatment for periodontitis, the second highest frequency of disease, and US $880 million were spent on these treatments. The annual direct per-capita medical costs of periodontitis were US $68 [14].

Risk factors of osteoporosis include female gender, increasing age, low body mass index (BMI), lifestyle factors (smoking, alcohol intake), menopause, and the use of medications including glucocorticoids, anticonvulsants and anticoagulants [15–19]. Osteoporosis and periodontitis share a common mechanism of action due to prostaglandins and proinflammatory cytokines [20, 21]. Periodontitis is regarded as a risk factor for comorbid diseases, such as cardiovascular and chronic inflammatory disease [22–24]. A recent large-scale cohort study identified a significant association between periodontitis and osteoporosis [12, 25]. Therefore, we hypothesized that periodontitis would be associated with the development of osteoporosis. The aim of this study was to investigate an association between osteoporosis and periodontitis using a representative population-based cohort. Gender-specific analyses were conducted, and a number of potential confounding variables were examined.

## Methods

### Participants

This study used National Health Insurance Service (NHIS) cohort data released in Korea. The dataset was comprised of 1,025,340 nationally representative participants and employed stratified sampling by gender, age and income level. The participants accounted for approximately 2.2% of the Korean population in 2002. The dataset also includes information on all medical claims filed and checkups attended by participants from 2002 to 2013.

Participants who were under 30 years of age, had been diagnosed with both osteoporosis in 2002 ~ 2004 and periodontitis in 2002 ~ 2003, and had no data related to a checkup in 2002 ~ 2003 were excluded from our analyses. Case and control groups were matched for gender, age, household income, type of Social Security, disability, and residential area using propensity score matching (PSM). The final sample included 13,464 participants: 8884 males and 4580 females. The case group included patients who were diagnosed with periodontitis. A statistical matching technique named PSM was conducted to reduce the bias due to confounding variables between the case and control group.

### Definition of periodontitis

Patients were defined as having periodontitis as per the 2004 International Classification of Disease 10th (ICD-10), code K05.3-K05.6 and been treated with subgingival curettage, root conditioning, periodontal flap operation, bone graft for alveolar bone defects, and guided tissue regeneration (Prescription code of NHIS: U1051–1052 Periodontal flap operation [Simple/complicated], U1071–1072 bone graft for alveolar bone defects [allogenic, xenogenous or substitute bone graft/autogenous bone graft], U1081–1083 guided tissue regeneration [without bone graft/allogenic, xenogenous or substitute bone graft/autogenous bone graft]).

### Definition of osteoporosis

The diagnosis of osteoporosis was the dependent variable. According to the ICD-10, osteoporosis was designated with main codes M80–M82. The different types of osteoporosis include osteoporosis with pathological fracture (M80), osteoporosis without pathological fracture (M81) and osteoporosis in diseases classified elsewhere (M82). Patients with osteoporosis were defined as those who visited clinics or hospitals at least two times per year for the condition and underwent bone densitometry.

### Confounding variables

Confounding variables included gender, baseline age, type of Social Security, income level, disability, residential area, Charlson Comorbidity Index (CCI), hypertension and diabetes mellitus, BMI, smoking, alcohol, and physical activity. Age was divided into four groups by 10-year periods (30–39, 40–49, 50–59, and 60+ years). The type of Social Security consisted of medical aid and health insurance in Korea. Health insurance was divided into industrial worker (IW) and self-employer (SE). The participants’ income level was recoded into five categories, from quintile 1 (low) to quintile 5 (high), according to the insurance premiums of all people in the household. The disability category was divided into normal and handicapped groups. The residential area was divided into either urban or rural. The CCI was calculated as the sum of the score of comorbid conditions (out of 22 total conditions). Each condition was assigned a score of 1, 2, 3, or 6. The CCI was coded again into three categories (0, 1, ≥2) [26, 27]. The hypertension and diabetes mellitus patients were participants who had been diagnosed with I10-I15 and E10-E14 in 2003. BMI was calculated as the patient’s weight in kilograms divided by the square of their height in meters. BMI was categorized as underweight (under 18.5 kg/m^2^), normal weight (18.5–23 kg/m^2^), overweight (23–27.5 kg/m^2^), and obese (over 27.5 kg/m^2^) [28]. Participants were classified into non-smoker, ex-smoker and smoker groups. The alcohol frequency was divided into none, 2–3/month, 1–2/week, 3–4/week, and ≥5/week. Physical activity was divided into none, 1–2/week, 3–4/week, 5–6/week, and 7/week according to the participants’ weekly frequency.

### Statistical analysis

All statistical analyses were conducted separately for each gender. We compared independent variables using x^2^-test according to the presence of periodontitis. The results were expressed as frequency (%). A Cox proportional hazard model was created to examine the differences in the development of osteoporosis between the study and the control group. Covariates included baseline age (ref: 30–39 years old), income level (ref: 1 quintile), type of Social Security (ref: Health insurance (IW)), disability (ref: normal), residential area (ref: urban), smoking (ref: non-smoker), alcohol (ref: none), physical activity (ref: none), BMI (ref: 18.5–23 kg/m^2^), CCI (ref: 0), hypertension (ref: non-diagnosed), and diabetes mellitus (ref: non-diagnosed). The overall incidence rate by age group (30–49, 50+) was calculated using a Kaplan-Meier curve for the 11–year follow-up period. The survival time was the number of months between the baseline and when osteoporosis was identified. The SAS statistical package version 9.2 was used to perform the analyses in this study. A *p*-value <0.05 was considered to be significant.

## Results

Table 1 shows the characteristics of the case and control group in males. No significant difference in gender, age, household income, type of Social Security, disability, or residential area between the case and control groups were identified because these variables were used for matching. The rates of overweight (BMI: 23–27.5 kg/m^2^) and obesity (BMI: > 27.5 kg/m^2^) in the case group were significantly higher than in the control group. Osteoporosis in males developed at a rate of 1.1% over 11 years. The average duration of osteoporosis at diagnosis was 10.79 years in patients with periodontitis which was lower than the duration in participants without periodontitis (10.83 years) (data not shown). Table 2 shows the characteristics of the case and control groups in females. The case group was more likely to experience diabetes than the control group. Osteoporosis in females developed at a rate of 15.8% over 11 years. The average duration of osteoporosis at diagnosis was 9.37 years in patients with periodontitis which was significantly lower than in participants without periodontitis (9.67 years, data not shown).

**Table 1:** Characteristics of the subjects according to diagnosis of periodontitis (male)

		Total		Non-Diagnosed		Diagnosed		
		*N*	%	*N*	%	*N*	%	*P*-value
Total		8884	100	7403	83.3	1481	16.7	
Age	30 ~ 39	2592	29.2	2160	83.3	432	16.7	1.0000
	40 ~ 49	3412	38.4	2843	83.3	569	16.7	
	50 ~ 59	1860	20.9	1550	83.3	310	16.7	
	≥ 60	1020	11.5	850	83.3	170	16.7	
Smoking	Non-smoker	3560	40.1	2992	84.0	568	16.0	0.2452
	Ex-smoker	773	8.7	648	83.8	125	16.2	
	Smoker	4551	51.2	3763	82.7	788	17.3	
Alcohol	None	2757	31.0	2286	82.9	471	17.1	0.8023
	2 ~ 3/month	2049	23.1	1716	83.7	333	16.3	
	1 ~ 2/week	2612	29.4	2183	83.6	429	16.4	
	3 ~ 4/week	998	11.2	823	82.5	175	17.5	
	≥ 5/week	468	5.3	395	84.4	73	15.6	
Physical activity	None	4253	47.9	3563	83.8	690	16.2	0.4111
	1 ~ 2/week	2920	32.9	2434	83.4	486	16.6	
	3 ~ 4/week	993	11.2	810	81.6	183	18.4	
	5 ~ 7/week	718	8.1	596	83.0	122	17.0	
BMI(kg/m^2^)	< 18.5	180	2.0	160	88.9	20	11.1	0.0001
	18.5 ~ 23	2990	33.7	2545	85.1	445	14.9	
	23 ~ 27.5	4763	53.6	3938	82.7	825	17.3	
	> 27.5	951	10.7	760	79.9	191	20.1	
CCI	0	6310	71.0	5262	83.4	1048	16.6	0.9692
	1	1691	19.0	1407	83.2	284	16.8	
	≥ 2	883	9.9	734	83.1	149	16.9	
Hypertension	NO	8277	93.2	6897	83.3	1380	16.7	0.9829
	YES	607	6.8	506	83.4	101	16.6	
Diabetes	NO	8568	96.4	7141	83.3	1427	16.7	0.8391
	YES	316	3.6	262	82.9	54	17.1	
Osteoporosis	NO	8787	98.9	7326	83.4	1461	16.6	0.2942
	YES	97	1.1	77	79.4	20	20.6	

*IW* industrial worker, *SE* self-employer, *BMI* body mass index, *CCI* Charlson comorbidity index

**Table 2:** Characteristics of the subjects according to diagnosis of periodontitis (female)

		Total		Non-Diagnosed		Diagnosed		
		*N*	%	*N*	%	*N*	%	*P*-value
Total		4580	100	3817	83.3	763	16.7	
Age	30 ~ 39	672	14.7	560	83.3	112	16.7	1.0000
	40 ~ 49	1926	42.1	1605	83.3	321	16.7	
	50 ~ 59	1218	26.6	1015	83.3	203	16.7	
	≥ 60	764	16.7	637	83.4	127	16.6	
Smoking	Non-smoker	4442	97.0	3705	83.4	737	16.6	0.6548
	Ex-smoker	12	0.3	9	75.0	3	25.0	
	Smoker	126	2.8	103	81.7	23	18.3	
Alcohol	None	3671	80.2	3025	82.4	646	17.6	0.0174
	2 ~ 3/month	568	12.4	497	87.5	71	12.5	
	1 ~ 2/week	251	5.5	218	86.9	33	13.1	
	3 ~ 4/week	60	1.3	51	85.0	9	15.0	
	≥ 5/week	30	0.7	26	86.7	4	13.3	
Physical activity	None	2992	65.3	2526	84.4	466	15.6	0.0446
	1 ~ 2/week	831	18.1	678	81.6	153	18.4	
	3 ~ 4/week	374	8.2	307	82.1	67	17.9	
	5 ~ 7/week	383	8.4	306	79.9	77	20.1	
BMI(kg/m^2^)	< 18.5	142	3.1	120	84.5	22	15.5	0.8475
	18.5 ~ 23	1922	42.0	1602	83.4	320	16.6	
	23 ~ 27.5	2026	44.2	1693	83.6	333	16.4	
	> 27.5	490	10.7	402	82.0	88	18.0	
CCI	0	2818	61.5	2370	84.1	448	15.9	0.074
	1	1090	23.8	884	81.1	206	18.9	
	≥ 2	672	14.7	563	83.8	109	16.2	
Hypertension	NO	4099	89.5	3420	83.4	679	16.6	0.6168
	YES	481	10.5	397	82.5	84	17.5	
Diabetes	NO	4407	96.2	3683	83.6	724	16.4	0.0342
	YES	173	3.8	134	77.5	39	22.5	
Osteoporosis	NO	3858	84.2	3233	83.8	625	16.2	0.0538
	YES	722	15.8	584	80.9	138	19.1	

*IW* industrial worker, *SE* self-employer, *BMI* body mass index, *CCI* Charlson comorbidity index

Table 3 contains the HRs for osteoporosis during an 11-year follow-up period using multivariate Cox proportional regression between males and females. In males, periodontitis was not associated with the development of osteoporosis. The risk factors for osteoporosis were increasing age and CCI score. In females, the risk factors of osteoporosis were increasing age, BMI, CCI score, diabetes, and periodontitis. Females with periodontitis were more likely to experience osteoporosis (HR: 1.22, 95% CI: 1.01–1.48). Women classified as overweight (BMI: 23–27.5 kg/m^2^) and obese (BMI: > 27.5 kg/m^2^) were also more likely to develop osteoporosis than their normal counterparts, as were those who had been diagnosed with diabetes (HR: 1.46, 95% CI: 1.01–1.48).

**Table 3:** Multivariate Cox proportional regression of risk factor of osteoporosis between male and female

		Male			Female		
		HR	95% CI	*P*-value	HR	95% CI	*P*-value
Age	30 ~ 39 (ref)	1			1		
	40 ~ 49	4.32	0.96–19.43	0.057	8.49	4.34–16.60	<.0001
	50 ~ 59	22.99	5.44–97.26	<.0001	22.62	11.57–44.20	<.0001
	≥ 60	45.14	10.60–192.32	<.0001	29.47	15.0–57.90	<.0001
Smoking	non-smoker (ref)	1			1		
	ex-smoker	0.58	0.21–1.63	0.301	1.59	0.22–11.45	0.646
	smoker	1.28	0.82–1.98	0.273	0.71	0.43–1.18	0.189
Alcohol	None (ref)	1			1		
	2 ~ 3/month	0.77	0.40–1.46	0.418	0.78	0.59–1.03	0.079
	1 ~ 2/week	0.95	0.54–1.67	0.849	0.75	0.49–1.15	0.188
	3 ~ 4/week	0.93	0.46–1.89	0.838	0.50	0.19–1.33	0.164
	≥ 5/week	1.38	0.72–2.65	0.329	0.51	0.16–1.59	0.246
Physical activity	None (ref)	1			1		
	1 ~ 2/week	0.63	0.37–1.08	0.092	0.83	0.67–1.03	0.082
	3 ~ 4/week	0.49	0.20–1.25	0.135	0.82	0.61–1.11	0.207
	5 ~ 7/week	0.75	0.40–1.43	0.380	1.02	0.79–1.31	0.894
BMI(kg/m^2^)	< 18.5	1.31	0.51–3.37	0.578	0.89	0.55–1.43	0.632
	18.5 ~ 23 (ref)	1			1		
	23 ~ 27.5	0.92	0.60–1.42	0.720	0.74	0.63–0.87	0.000
	> 27.5	0.33	0.10–1.08	0.066	0.59	0.45–0.77	<.0001
CCI	0 (ref)	1			1		
	1	1.89	1.17–3.05	0.010	1.20	1.00–1.43	0.046
	≥ 2	2.25	1.34–3.78	0.002	1.36	1.11–1.66	0.003
Hypertension	NO (ref)	1			1		
	YES	1.21	0.63–2.31	0.569	1.03	0.83–1.28	0.807
Diabetes	NO (ref)	1			1		
	YES	1.65	0.65–4.15	0.290	1.46	1.02–2.10	0.041
Periodontitis	NO (ref)	1			1		
	YES	1.39	0.85–2.29	0.190	1.22	1.01–1.48	0.038

*IW* industrial worker, *SE* self-employer, *BMI* body mass index, *CCI* Charlson comorbidity index

**Fig. 1:**
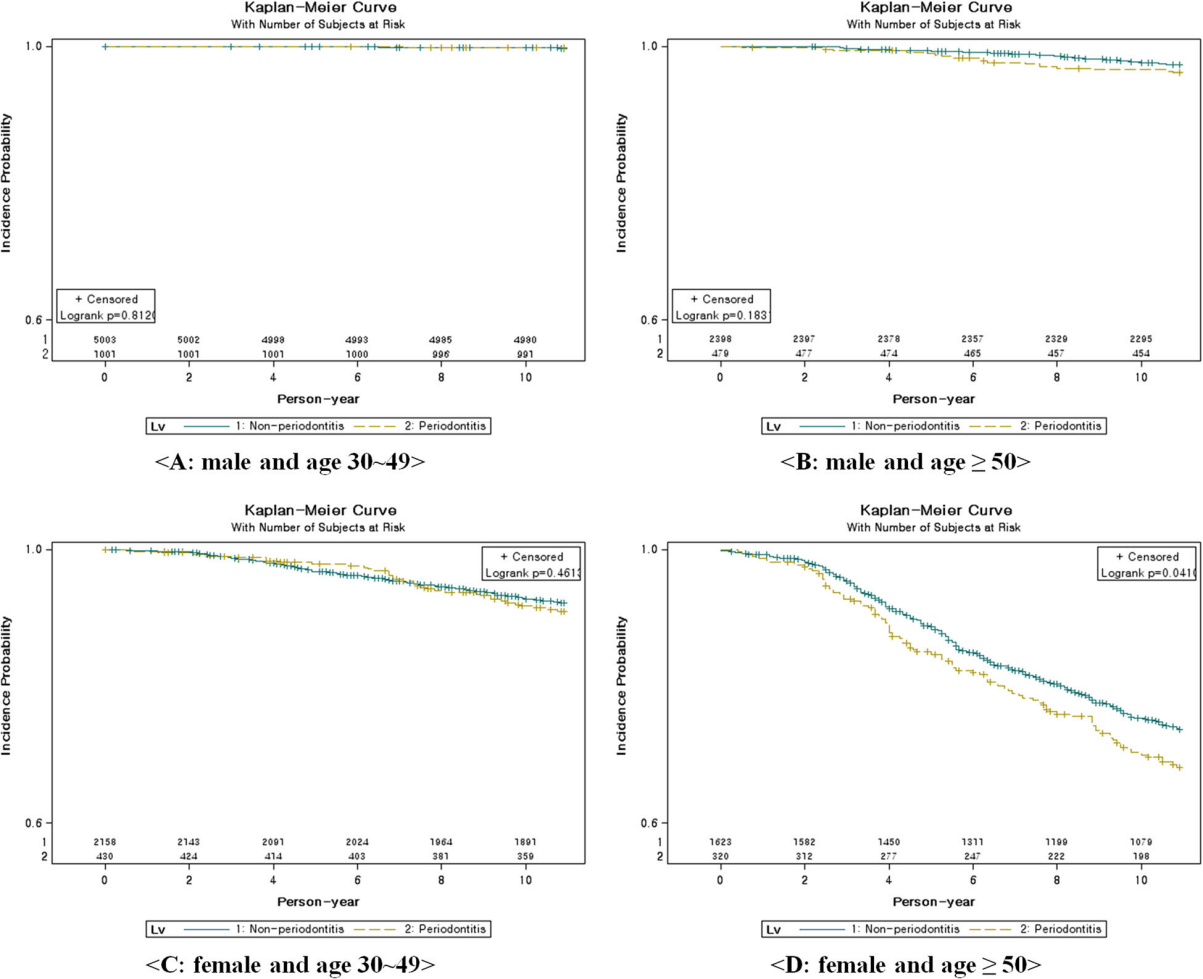
Kaplan-Meier curves of subjects by gender and age-group over 11 years, according to presence of periodontitis

Figure 1 shows the curves that illustrate the incidence probability over an 11-year follow-up period. A subgroup analysis where gender and age (30–49, ≥ 50) were stratified into four groups using a Kaplan-Meier curve was conducted. The females experienced osteoporosis more frequently than males. In particular, females over the age of 50 were diagnosed with osteoporosis more frequently than females aged 30–49 years (Fig. 1c, d). The incidence of osteoporosis in participants with periodontitis was higher in this group than in any other.

## Discussion

Studies on the incidence and prevalence of osteoporosis have revealed various details depending on the gender and age of the participants. In 2012, there were 4120 and 1165 patients per 100,000 in the entire Korean population who were newly diagnosed with and treated for osteoporosis [29]. In a previous Korean-oriented study, the prevalence of osteoporosis in participants over 40 and under 79 was 13.1% in males and 24.3% in females according to the World Health Organization (WHO) criteria [4]. In a different study, the prevalence of osteoporosis in participants aged over 50 years was 35.5% in women and 7.5% in men [30]. Our study also showed a female gender tendency and dependency on age. According to these results, the crude incidence of osteoporosis was 1.1% in males and 15.8% in females over an 11-year period. In females, the average duration of osteoporosis at diagnosis for patients with periodontitis was significantly lower than the duration in patients without periodontitis.

The risk factors for osteoporosis are increasing age, BMI, CCI score, and periodontitis. Our findings have revealed that there is a higher risk of osteoporosis in females than in males. The gap between males and females widens after participants reach their 40s because females experience estrogen deficiency during post-menopause [31]. An increasing age is also associated with a higher risk of osteoporosis. Estrogen and serum total testosterone levels decrease with age [32–34]. Females with a normal BMI have a significantly higher risk of osteoporosis compared with females who are overweight and obese. Participants with a higher BMI also tend to reach a higher peak bone mineral density than those with a lower BMI [35]. Comorbidities as measured by the CCI score have been associated with an increase in disease incidence. Both osteoporosis and the CCI score had a strong positive relationship [36]. The HR for the osteoporosis of subjects with CCI 1 or ≥2 was also higher than for participants with a CCI of 0. CCI score therefore has a strong influence on the development of osteoporosis in males. Lifestyle factors, such as smoking, drinking alcohol and level of physical activity, were not associated with the development of osteoporosis. However, diabetes was related with the development of osteoporosis in females. Patients with diabetes may have an increased risk for osteoporosis. Bone and mineral abnormalities in patients with diabetes might be caused by the effects of insulin deficiency or resistance and hyperglycemia on the bone and bone marrow microenvironments [37]. Males with periodontitis were not significantly associated with the development of osteoporosis. In contrast, females who had been diagnosed with periodontitis were significantly likely to go on to develop osteoporosis. In particular, females aged ≥50 with periodontitis experienced a higher rate of osteoporosis than others.

Studies to assess the extent of the relationship between periodontitis and osteoporosis are ongoing. The findings of most studies have revealed that the reasons for the connection between the two disorders are estrogen deficiency and low bone mineral density. According to a systematic review on the relationship between periodontitis and osteoporosis, the diagnostic criteria for periodontitis serve an important function. In studies that used radiological criteria to define periodontitis, the relationship between periodontitis and osteoporosis is usually positive. However, in those investigations that used clinical criteria to define periodontitis, the results are controversial [38].

The severity of periodontitis is quickly becoming an indicator for the presence of osteoporosis elsewhere in the body, since osteoporosis associated with periodontitis is known to worsen the severity of bone degradation in the mouth. Early detection of this condition allows for more adequate treatment of osteoporosis before it has the chance to cause debilitating fractures.

The possibility of bias may arise because the obvious differences in outcome between two groups might depend on intrinsic characteristics. In randomized experimental studies, the randomization enables unbiased estimation; for each covariate, randomization implies that the case groups will be balanced on average, according to the law of large numbers [39]. However, in observational studies, the assignment of research participants is typically not randomized. Instead, PSM attempts to mimic randomization by creating a case group that is comparable on all observed covariates to a control group.

This study had the following limitations. First, the data used in our analysis were claim data and therefore only included information on each episode of health care utilization. These claim data do not contain any clinical findings or information about the disease’s severity. Patients with osteoporosis were defined by examining the results of bone densitometry regardless of any other clinical findings. Second, important confounding variables, including menopause, medications, dietary factors, and metabolic syndrome, associated with osteoporosis were not available for the cohort and therefore limit the value of these findings. Previous studies have revealed the effect of menopause, medication, dietary habits, and metabolic syndrome on osteoporosis [15–17, 40, 41]. Despite these limitations, a key advantage of this study was its demonstration of an association between periodontitis and osteoporosis using 11 years of representative, population-based follow-up data. Third, claim-based diagnoses might underestimate the real prevalence or incidence of periodontitis and osteoporosis.

## Conclusion

The evidence for a link between periodontitis and osteoporosis remains conflicted, so this study was conducted to further investigate this association. We found that periodontitis seems to be associated with the development of osteoporosis after matching participants for gender, age, household income, type of Social Security, disability, and residential area. In particular, females aged ≥50 with periodontitis experience a higher rate of osteoporosis. To both delay and prevent osteoporosis, it is necessary for the good management of teeth. This includes dental examinations, regular cleanings and gum treatment.

## Abbreviations

BMI: Body mass index
CCI: Charlson comorbidity Index
ICD: International classification of disease
IW: Industrial worker
KCD: Korean classification of diseases
NHIS: National Health Insurance Service
PSM: Propensity score matching
SE: Self-employer
WHO: World Health Organization
